# Literature review and case study of predominant tubulointerstitial lupus nephritis

**DOI:** 10.1080/0886022X.2025.2558089

**Published:** 2025-09-18

**Authors:** Jun Wang, Ying Zuo, Chunqiu Liu, Mengni Ke, Yuewu Tang, Lei Liu

**Affiliations:** ^a^ Department of Nephrology, Chongqing University Three Gorges Hospital, Chongqing, China; ^b^ Department of Nephrology, Chongqing Three Gorges Central Hospital, Chongqing, China

**Keywords:** Systemic lupus erythematosus (SLE), lupus nephritis (LN), tubulointerstitial nephritis, pathology

## Abstract

**Background:**

Systemic lupus erythematosus (SLE) is a chronic inflammatory disease that affects multiple organ systems, and lupus nephritis (LN) primarily involves glomerular, vascular and tubulointerstitial lesions. Renal tubulointerstitial lesions are almost always present alongside glomerular lesions, with isolated renal tubulointerstitial LN being very rare. The aim of this study was to evaluate the clinicopathological features, differential diagnosis, treatment and prognosis of predominant tubulointerstitial lupus nephritis (PTILN).

**Case presentation:**

We present the case of a 64-year-old Chinese male with severe SLE. The diagnosis of SLE was confirmed on the basis of proteinuria, positive antinuclear antibodies, and immunological and haematological disorders. Renal biopsy revealed severe chronic tubulointerstitial nephritis with excessive immune complex deposition in the tubular basement membrane (TBM), mild glomerular lesions and minimal immune complex deposition, suggesting predominant tubulointerstitial LN. The patient was treated with steroids and mycophenolate mofetil, leading to stabilization of renal function. In the literature review, we identified a total of 18 patients whose predominant tubulointerstitial LN was reported. In our analysis, we evaluated the clinical and pathological features, treatment, and prognosis of this condition in these patients.

**Conclusions:**

This case series reinforces PTILN as a distinct LN variant characterized by dominant tubulointerstitial inflammation and TBM immune deposits. Although PTILN shares clinical features with classic LN, PTILN exhibits notable heterogeneity in demographics, pathology, and outcomes, and its pathogenesis, diagnostic criteria, and optimal management require further elucidation.

## 1. Background

Lupus nephritis (LN) is a common manifestation of systemic lupus erythematosus (SLE) that can result in irreversible renal damage. In addition to glomerular injury, tubulointerstitial lesions are also a notable feature of LN [1]. Previous investigations have demonstrated that most LN patients have renal tubulointerstitial abnormalities [2,3]. Moreover, tubulointerstitial markers constitute significant independent risk factors for renal outcomes in patients with LN [2–5]. The 2018 revision of the International Society of Nephrology/Renal Pathology Society (ISN/RPS) classification system for LN emphasized the importance of tubulointerstitial lesions [6]. Although most cases involve primarily glomerular pathology, the predominance or exclusive presence of tubulointerstitial alterations, especially in SLE patients with minimal glomerular abnormalities, is infrequent [7]. Our literature review identified only 18 such cases. In the present study, we present a case of predominant tubulointerstitial lupus nephritis (PTILN) and review the clinical and pathological features, treatment, and prognosis of this rare condition.

## 2. Case presentation

### 2.1. Clinical features and laboratory findings

On 23 July 2022, a 64-year-old Chinese male was admitted to our hospital due to a two-month history of fatigue, reduced appetite and hematemesis persisting for three days. Approximately two months earlier, he initially reported weakness in his lower limbs, loss of appetite, frothy urine and increased nocturnal urination frequency (3–4 times/night), without noticeable lower limb edema; however, he did not take these symptoms seriously. Despite seeking medical attention, lower limb fatigue and decreased appetite persisted. Three days prior to admission, he experienced unexplained vomiting of blood, which initially contained coffee-ground-like substances of unknown volume, followed by an episode of vomiting dark red liquid with blood clots (approximately 250 mL) accompanied by dizziness.

The patient had previously sought medical care at a local hospital, where blood tests revealed a hemoglobin level of 46 g/L, a serum urea nitrogen level of 36.23 mmol/L, a serum creatinine (SCr) level of 866 μmol/L and a potassium level of 6.4 mmol/L. The preliminary diagnosis included upper GI bleeding, severe anemia and acute kidney injury. Following symptomatic treatments such as fluid replacement, red blood cell transfusion, acid suppression and haemostasis, vomiting ceased, and the patient was subsequently transferred to our hospital for further evaluation and treatment.

The patient had a history of chronic gastritis for over 10 years. In 2022, gastroscopy revealed exposed gastric veins and confirmed the presence of chronic gastritis. Slightly over a year ago, he was admitted to the hospital due to acute cholecystitis associated with gallstones and acute pancreatitis, and during admission, his hemoglobin level was 138 g/L, and his creatinine level was 62 μmol/L. There was no history of exposure to specific drugs or toxins, long-term usage of nonsteroidal anti-inflammatory drugs (NSAIDs), Chinese herbal medicine or any other medications known to induce renal tubulointerstitial lesions. His family medical history did not contribute to his current condition.

Upon admission, the patient’s physical examination revealed a blood pressure of 126/70 mmHg, a temperature of 36.5 °C, a pulse rate of 63 beats/min, and a respiratory rate of 18 breaths/min. He exhibited signs of anemia with pale palpebral conjunctiva, no dental abnormalities, no abdominal tenderness, and no notable edema in the lower limbs.

The patients’ laboratory results are presented in Supplementary Table 1. Cardiac ultrasound indicated mild aortic regurgitation but normal overall systolic function of the left ventricle. Color Doppler ultrasound of the kidneys and blood vessels revealed a normal kidney size with an elevated renal artery resistivity index. Gastroscopy suggested acute erosive hemorrhagic gastritis as a probable cause of upper GI bleeding, and a subsequent colonoscopy revealed no significant abnormalities in the intestinal mucosa.

On the basis of the 2019 European League Against Rheumatism (EULAR)/American College of Rheumatology (ACR) classification criteria for SLE, the patient’s results, which included a positive ANA > 1:80 according to the HEp-2 method (1:1000 nuclear dots), met the initial criterion. The laboratory results included leukopenia (3 points), thrombocytopenia (4 points), proteinuria > 0.5 g/24 h (4 points), low C3 and C4 levels (4 points), and the presence of anti-dsDNA antibodies (6 points), resulting in a total score of 18 points (per EULAR/ACR guidelines, only the highest-weighted item within a domain is counted (here, thrombocytopenia [4 points] > leukopenia [3 points]). This score supports the classification of SLE, fulfilling the requirement of ≥10 points and at least one clinical criterion, thereby suggesting a diagnosis of SLE in this patient. The clinical diagnosis of SLE was established on the basis of multisystem involvement (renal, haematological), immunological dysregulation (hypocomplementemia, autoantibodies), and the exclusion of competing diagnoses.

### 2.2. Renal biopsy

**Figure 1.**
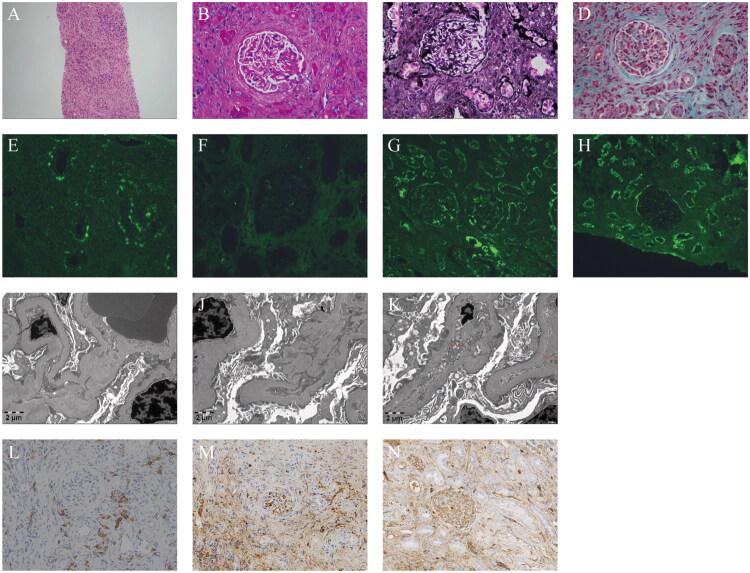
Renal biopsy findings of the SLE patient. (A–D) Light microscopy images showing mild glomerular lesions, granular degeneration of renal tubular epithelial cells, diffuse atrophy, and extensive interstitial inflammatory cell infiltration accompanied by fibrosis. (A) Hematoxylin and eosin (HE) staining, magnification ×100. (B) Periodic acid-Schiff (PAS) staining, magnification ×400. (C) Periodic acid-silver methenamine (PASM) staining, magnification ×400. (D) Masson’s trichrome staining, magnification ×400. (E–H) Immunofluorescence staining results: (E) Granular deposition of IgG observed along the tubular basement membrane (TBM) and in the interstitium, magnification ×400; (F) Slight deposition of IgA in the glomerular mesangial area and TBM, magnification ×400; (G) Diffuse or segmental deposition of C3 in the glomerular mesangial area and Bowman’s capsule, with linear deposition along the TBM, magnification ×200, and; (H) Diffuse or segmental deposition of C1q in the glomerular mesangial area and Bowman’s capsule, with linear deposition along the TBM, magnification ×200. (I–K) Electron microscopy images depicting deposition of electron-dense material in the mesangial area, diffuse renal tubular atrophy, and deposition of electron-dense material in the TBM. Interstitial inflammatory cell infiltration with collagen fiber proliferation, magnification ×8,000. (L–N) Immunohistochemical staining for CD38, IgG, and IgG4, magnification ×200.

Percutaneous renal biopsy was conducted, and the obtained samples were subjected to hematoxylin and eosin (HE), periodic acid–Schiff (PAS), periodic acid–silver methenamine (PASM) and Masson staining. Light microscopy revealed predominantly renal cortex tissues with 12 visible glomeruli, an absence of glomerular or segmental sclerosis, no significant proliferation of glomerular mesangial cells or matrix, and only slight fuchsin deposition in the mesangial area. Additionally, there were open capillary loops and a normal-thickness glomerular basement membrane (GBM), whereas nail processes, double track changes, mesangial insertion, subepithelial or subendothelial fuchsin deposition, platinum ear-like structures, proliferative wall epithelial cells, and crescent bodies were absent. In the renal interstitium, the findings included granular degeneration of renal tubular epithelial cells, diffuse atrophy and diffuse inflammatory cell infiltration with fibrosis, along with thickening of the small arterial wall, intimal fibrous tissue proliferation, and lumen stenosis (Figure 1A–D). Immunofluorescence analysis revealed substantial deposition of multiple immune complexes (IgG, C3, C1q, kappa, and lambda) in the tubular basement membrane (TBM) and the mesangial area of the glomerulus, Bowman’s capsule walls, and small arterial walls (Figure 1E–H). Electron microscopy revealed mild homogeneous thickening of the GBM, extensive fusion of foot processes, diffuse tubular atrophy and electron-dense substance deposition in the mesangial area and TBM. Renal interstitial inflammation with infiltrating inflammatory cells and collagen fiber proliferation was also evident (Figure 1I–K). Immunohistochemical investigations revealed multifocal infiltration of CD38-positive cells in the renal interstitium (Figure 1L), partial IgG cell positivity (Figure 1M), scattered IgG4 cell positivity (Figure 1N), and an IgG4+/IgG + ratio of <40%, with some fields exhibiting more than 10 IgG4-positive cells. On the basis of these observations, the diagnosis of predominant tubulointerstitial LN was confirmed.

### 2.3. Treatment and follow-up

**Figure 2.**
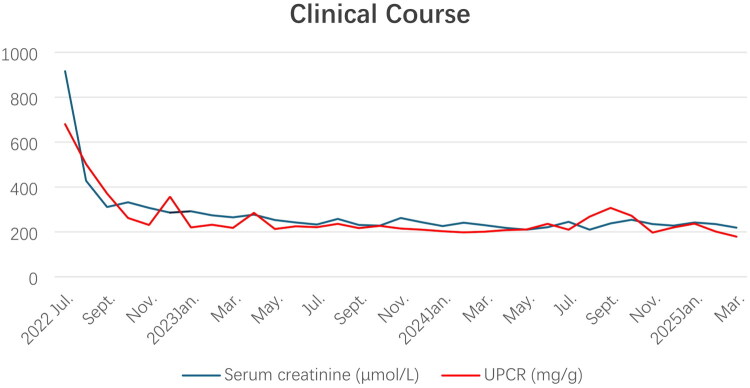
Clinical course of the patient’s serum creatinine and UPCR levels.

After admission, the patient received symptomatic treatment, including acid suppression and haemostasis to manage GI bleeding, along with hemodialysis therapy. After confirmation of the SLE diagnosis, immediate administration of methylprednisolone was initiated at a daily dosage of 250 mg for 3 days, followed by a continuous intravenous dose of 40 mg daily. This treatment was complemented with the oral administration of hydroxychloroquine sulfate at 400 mg/day and mycophenolate mofetil (MMF) at 2 g/day. After one month, the patient’s serum creatinine levels stabilized within the range of 250–350 µmol/L, allowing for the complete discontinuation of dialysis. Three months into the treatment, the urinary protein-to-creatinine ratio (UPCR) decreased to 262 mg/g Cr, and the dose of methylprednisolone was gradually tapered to 4 mg daily. After 6 months, the amount of MMF decreased to 1.5 g/day. Throughout the subsequent two years of follow-up, the patient’s condition remained stable, with serum creatinine levels ranging consistently from 200 to 300 µmol/L (Figure 2 and Supplementary Table 2).

## 3. Literature review

### 3.1. Methods

#### 3.1.1. Information sources

In this study, we conducted a systematic search of the PubMed, Web of Science, EMBASE, MEDLINE (Ovid), and Directory of Open Access Journals (DOAJ) databases using the combination of keywords ‘Systemic Lupus Erythematosus’ or ‘Lupus Nephritis’ and ‘Tubulointerstitial Nephritis’. No restrictions were placed on publication date to ensure that the comprehensive retrieval of articles

#### 3.1.2. Literature inclusion and exclusion criteria

The inclusion criteria were as follows: case reports or case series with detailed clinical data and extractable complete case information. The reported case had a confirmed diagnosis of PTILN on the basis of renal biopsy findings, characterized by dominant tubulointerstitial inflammation and/or fibrosis with only mild or no glomerular involvement. The patient fulfilled the 2019 EULAR/ACR classification criteria for SLE, with a total score of 10 or more.

The exclusion criteria were as follows: repeated reports, animal experiments, clinical trials and secondary analysis of literature; literature in which the original text or full text could not be found; incomplete medical history information of reported cases; and cases with overlapping autoimmune disorders such as IgG4-related disease (IgG4-RD) or primary Sjögren’s syndrome, supported by clinical, serological, or histological findings.

#### 3.1.3. Literature screening

**Figure 3.**
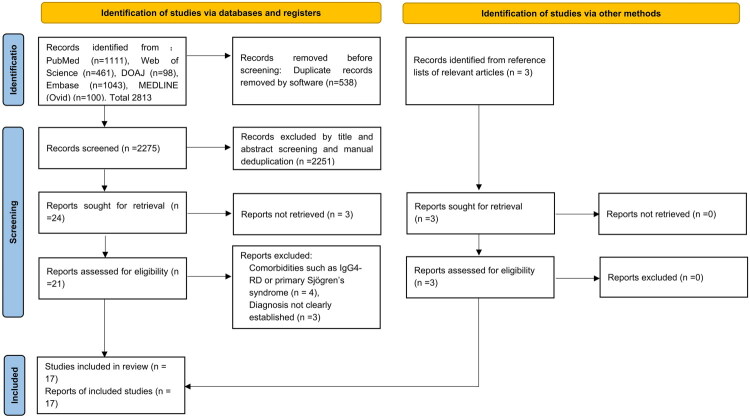
Article selection process flowchart (based on PRISMA).

Our systematic search identified 2,813 records from five databases. After 538 duplicates were removed, 2,275 unique records were subjected to title/abstract screening. Of these, 2,251 were excluded, leaving 24 articles for full-text assessment. Three full-text articles were unavailable, leaving 21 articles for detailed evaluation. Following the application of the inclusion/exclusion criteria, seven studies were excluded, including four due to comorbidities (e.g., IgG4-RD/Sjögren’s syndrome) and three with unclear diagnoses. Additionally, we screened the references of relevant full-text articles and identified three additional eligible studies. Thus, 17 studies (reporting 18 cases) met the inclusion criteria. A total of 17 articles (involving 18 patients), including our case (19 patients in total), were ultimately included in this study. A flow chart describing the reference selection process is illustrated in Figure 3.

#### 3.1.4. Data extraction

Patient information was gathered by thoroughly reviewing the full text, which included details such as the publication year, patient’s sex, age, presenting symptoms, abnormal test findings, histopathological features, administered therapeutic interventions and prognosis. Each patient was individually scored according to the 2019 EULAR/ACR classification criteria for SLE. Data extraction and sorting were performed using Microsoft Excel 2019 (Microsoft Corporation, USA). In this study, we evaluated the clinical and pathological features, treatment, and prognosis of this condition in these patients (Tables 1–3; Supplementary Tables 3 and 4).

**Table 1.** Demographics and clinical features of 19 patients with PTILN.

Variable	Values	Available cases (*n*)
Number of patients	19	19
Age	42.0 (3.0–72.0) IQR: 39.0	19
Aged ≥60 years (*n*, %)	7 (36.8%)	19
Sex (F:M)	9:10	19
Race/Ethnicity		19
Black (*n*, %)	3 (15.8%)	
White (*n*, %)	3 (15.8%)	
Asian (*n*, %)	3 (15.8%)	
Unknow (*n*, %)	10 (52.6%)	
SLE disease duration (month)	6.0 (2.0,19.0)	17
Serum creatinine (mg/dL)	2.7 (1.47,5.33)	17
eGFR (ml/min/1.73 m²)	20.27 (10.61,54.86)	18
24h urinary protein (g)	0.76 (0.24,1.05)	10
Acute kidney injury (*n*, %)	11 (57.9%)	19
Nephrotic syndrome (*n*, %)	1 (5.3%)	19
Active urinary sediment (*n*, %)	8 (50%)	16
Fever (*n*, %)	8 (57.1%)	14
Hematologic (*n*, %)	17 (100%)	17
Neuropsychiatric (*n*, %)	2 (40%)	5
Mucocutaneous (*n*, %)	10 (83.3%)	12
Serosal (*n*, %)	5 (62.5%)	8
Musculoskeletal (*n*, %)	13 (92.9%)	14
Complement (C3 or C4↓) (*n*, %)	14 (93.3%)	15
Anti-dsDNA+ (*n*, %)	16 (94.1%)	17
ANA+ (*n*, %)	18 (100%)	18

F: female; M: male; IQR: interquartile range; ↓ represents less than the reference range.

**Table 2.** Summary of histopathologic findings in 19 patients with PTILN.

Variable	Values [*n* (%)]	Available cases (*n*)
Number of patients	19	
Light Microscopy (LM)		
Interstitial Inflammation	17 (89.5%)	19
Tubular Atrophy (TA)	12 (63.2%)	19
Interstitial Fibrosis (IF)	13 (68.4%)	19
Tubular Atrophy > 50%	4 (33.3%)	12
Interstitial Fibrosis > 50%	4 (36.4%)	11
No or mild glomerular lesions	19 (100%)	19
Immunofluorescence (IF)		
TBM immune complex or (and) complement deposits	17 (94.4%)	18
TBM deposit components		
IgG	14 (93.3%)	15
IgA	3 (27.3%)	11
IgM	4 (40%)	10
C3	13 (86.7%)	15
C1q	11 (73.3%)	15
Mesangium immune complex or (and) complement deposits	9 (50%)	18
Electron Microscopy (EM)		
Electron-dense deposits in TBM	8 (61.5%)	13
Electron-dense deposits in mesangium	6 (46.2%)	13

**Table 3.** Treatment strategies, therapeutic response, and follow-up in 19 patients with PTILN.

No. of case	Corticosteroids	MMF	CTX	Other agents	Therapeutic response	Follow-up duration (months)
1 [8]	√	++++	–	AZA	PR	Not mentioned
2 [9]	√	–	–	–	CR	36
3 [10]	√	–	–	–	CR	6
4 [11]	√	–	–	–	PR	15
5 [11]	–	–	–	–	ESRD	3
6 [12]	√	–	√	–	Death (sepsis)	
7 [13]	√	–	–	–	CR	24
8 [14]	–	–	–	–	NR	36
9 [15]	√	–	–	–	PR	72
10 [16]	√	–	–	–	PR	90
11 [17]	√	–	–	–	CR	12
12 [18]	√	–	–	–	CR	16
13 [19]	Not mentioned					
14 [20]	√	–	–	AZA	CR	24
15 [21]	√	√	√	–	PR	10
16 [22]	√	–	–	–	PR	30
17 [7]	√	√	–	HCQ	PR	31
18 [23]	√	√	–	HCQ	PR	24
19(OUR CASE)	√	√	–	HCQ	PR	32

A checkmark (√) indicates the use of the corresponding medication; a dash (–) indicates no use. CR (complete response): normalization of serum creatinine (±15% of baseline) + proteinuria <0.5 g/day; PR (partial response): >50% improvement in serum creatinine but not normalized + proteinuria <1 g/day; NR (no response): failure to achieve PR; ESRD (end-stage renal disease): requirement for renal replacement therapy; death: mortality during follow-up; MMF: mycophenolate mixture; CTX: cyclophosphamide; AZA: azathioprine; HCQ: hydroxychloroquine.

### 3.2. Results

#### 3.2.1. Demographic and clinical characteristics

This analysis of 19 biopsy-confirmed PTILN patients (Table 1, Supplementary Tables 3 and 4) revealed distinct demographic and clinical patterns. The cohort had a median age of 42.0 years (IQR: 39.0; range 3–72), with 36.8% (7/19) aged ≥60 years. A nearly equal sex distribution (9 females, 10 males) contrasts with the 9:1 female predominance in classic lupus nephritis. Racial data were unavailable for 52.6% (10/19) of the patients; among the remaining patients, black, white, and Asian patients each accounted for 15.8% (3/19) of the sample.

Clinically, acute kidney injury (AKI) predominated (57.9%, 11/19), with severe renal impairment at presentation (median creatinine 2.7 mg/dL [IQR: 1.47–5.33]; median eGFR 20.27 mL/min/1.73 m^2^ [IQR: 10.61–54.86]). Proteinuria was typically subnephrotic (median 24-h urine protein 0.76 g [IQR: 0.24–1.05]), with nephrotic-range proteinuria noted in only 1 patient (patient 6, Supplementary Table 3). Active urinary sediment occurred in 50% (8/16) of the patients.

Systemic manifestations were pervasive: hematologic involvement (100% of 17 evaluated) included anemia (e.g., Hb 46 g/L in our patient), leukopenia (e.g., WBC 3.22 × 10^9^/L in our patient), and thrombocytopenia (e.g., platelets 56 × 10^9^/L in our patient). Mucocutaneous (83.3% of 12) and musculoskeletal (92.9% of 14) involvement was frequent, and serositis (62.5% of 8) and fever (57.1% of 14) were common. The immunological hallmarks included universal ANA positivity (100%), anti-dsDNA antibodies (94.1%), and hypocomplementemia (low C3/C4 in 93.3%). The median SLE duration before renal diagnosis was 6.0 months (IQR: 2.0–19.0), indicating rapid renal involvement. Please refer to Table 1 and Supplementary Table 4.

#### 3.2.2. Histopathological features

The histopathological characteristics of 19 biopsy-confirmed PTILN patients revealed distinct patterns.

##### 3.2.2.1. Light microscopy (LM) findings

All patients (19/19, 100%) presented minimal or mild glomerular lesions, with no significant proliferation, sclerosis, crescents, or endocapillary hypercellularity. Interstitial mononuclear cell infiltration was present in 89.5% (17/19) of the patients, typically involving lymphocytes, plasma cells, and occasional eosinophils. Tubular atrophy (TA) and interstitial fibrosis (IF) were prominent, affecting 63.2% (12/19) and 68.4% (13/19) of the patients, respectively. Notably, 33.3% (4/12) demonstrated TA involving >50% of the parenchyma, whereas 36.4% (4/11) showed severe interstitial fibrosis (IF) (>50% involvement). The lymphoid follicles (patient 16) and ‘storiform’ fibrosis (patient 17) were detected in select patients, although these conditions were uncommon. Please refer to Table 2 and Supplementary Table 3.

##### 3.2.2.2. Immunofluorescence (IF) findings

Immune complexes were detected along the TBM in 94.4% (17/18) of the patients (Table 2). Although only 2 patients’ immune complexes exhibited a ‘full-house’ pattern during TBM deposition, most (72.2%, 13/18) deposited at least two types of immune complexes during TBM (Supplementary Table 3). IgG (94.3%, 14/15), C3 (86.7%, 13/15), and C1q (73.3%, 11/15) were the dominant components. IgA (27.3%, 3/11) and IgM (40%, 4/10) positivity was less common (Table 2). Faint mesangial staining occurred in 50.0% (9/18) but was consistently milder than TBM involvement (Table 2).

##### 3.2.2.3. Electron microscopy (EM) findings

Electron-dense deposits (EDDs) were identified in the TBM in 61.5% (8/13) of the patients (Table 2; patients 1, 9, 11, 12, 16–19; Supplementary Table 3). Mesangial EDDs occurred in 46.2% (6/13) of the patients (Table 2; Patients 3, 7, 12, 15, 17, and 19; Supplementary Table 3).

#### 3.2.3. Treatment and prognosis

##### 3.2.3.1. Treatment strategies

Corticosteroids formed the backbone of therapy (88.9%, 16/18) and were used as monotherapy in 56.3% (9/16) of the patients (Table 3). For combination therapy, MMF was the most common adjunct (22.2%, 4/18), followed by cyclophosphamide (CTX; 11.1%, 2/18) and azathioprine (AZA; 11.1%, 2/18). Hydroxychloroquine (HCQ) was administered to 16.7% (3/18) of the patients, including our patient (patients 17–19, Table 3). Two patients (Patients 5 and 8) received only supportive treatment but did not use immunosuppression (Supplementary Table 3).

##### 3.2.3.2. Therapeutic response and follow-up

The overall response rate (CR+PR) was 83.3% (15/18), 33.3% (6/18) achieved complete remission (CR), and 50% (9/18) achieved partial remission (PR) (Table 3). One patient (patient 8) showed no response (NR) to supportive care. Two patients progressed to ESRD (patient 5: dialysis dependent; patient 6: died from sepsis during follow-up) (Supplementary Table 3).

MMF-containing regimens (patients 15, 17–19) achieved a PR in all 5 patients, with sustained renal stability over 10–32 months. Long-term stability was observed even in partial remission (e.g., patients 9–10 maintained stable renal function for 72–90 months) (Table 3 and Supplementary Table 3).

The follow-up duration ranged from 3 to 90 months (median: 24 months). Three patients lacked long-term data, limiting outcome assessment. Most patients (15/16) maintained stable renal function or achieved remission. Please refer to Table 3 and Supplementary Table 3.

## 4. Discussion

According to the 2004 ISN/RPS classification of LN (ISN/RPS 2004), LN is categorized into six classes: I (minimal mesangial), II (mesangial proliferative), III (focal proliferative), IV (diffuse proliferative), V (membranous), and VI (sclerosing) [6,24]. Renal tubulointerstitial lesions, which are frequently observed in LN, are increasingly acknowledged as robust predictors of adverse outcomes [2–4], emphasizing the importance of these lesions in LN. Nevertheless, significant or isolated tubulointerstitial damage is infrequent [7].

This report describes the case of a male Chinese patient diagnosed with severe chronic tubulointerstitial nephritis associated with SLE, characterized by substantial immune complex deposition in the renal tubular basement membrane. Sjögren syndrome (SS) is a well-recognized cause of interstitial nephritis. However, the patient did not present with dry mouth or dry eyes, and anti-SS-A/B antibodies and Schirmer’s test results were negative. In addition, no significant findings were noted on salivary scintigraphy, and the diagnosis of SS was ruled out. Despite testing positive for rheumatoid factor, the absence of symmetrical multijoint pain, morning stiffness and a negative result for anti-cyclic citrullinated peptide antibody excluded a diagnosis of rheumatoid arthritis.

The possibility of IgG4-RD was also considered. However, his immunohistochemical examination of the renal biopsy revealed partial IgG-positive cells, scattered IgG4-positive cells and an IgG4+/IgG + ratio of less than 40%. Although a few high-power fields displayed more than 10 IgG4+ cells, the absence of widespread or localized organ enlargement or masses, normal serum IgG4 levels and the lack of storiform fibrosis in renal biopsy samples did not align with a diagnosis of IgG4-RD.

Severe anemia and thrombocytopenia prompted the evaluation of Evans syndrome and thrombotic microangiopathy. Hemolytic workup revealed normal reticulocytes, LDH, bilirubin, and haptoglobin; direct Coombs testing was negative. A peripheral smear revealed no schistocytes. ADAMTS13 activity was normal, and antiphospholipid antibodies were negative. Platelet and hemoglobin stabilization after transfusions and immunosuppression support SLE-related cytopenias rather than thrombotic microangiopathy or Evans syndrome.

A comprehensive patient history inquiry revealed no use of nephrotoxic medications (such as NSAIDs or Chinese herbal medicines with unknown properties) or exposure to harmful environmental toxins (i.e., cadmium or lead). Consequently, the possibility of renal tubulointerstitial damage caused by drugs or toxins was excluded. Furthermore, there was no indication that infection, tumors, or metabolic factors play a role in the patient’s condition.

Finally, our patient was diagnosed with PTILN. Our systematic literature review identified 18 previously reported cases meeting our inclusion criteria. Combined with the present patient, this integrated cohort analysis comprises 19 biopsy-confirmed PTILN patients, representing the largest consolidated study of this rare variant to date. Our findings reveal distinct clinicopathological patterns that challenge the traditional paradigm of LN, demonstrating significant deviations from classic LN across three key domains: demographic distribution, histopathological characteristics, and treatment response patterns.

Strikingly, the demographic profile of PTILN patients diverges markedly from that of classic lupus nephritis patients. Although classic LN exhibits a well-established female predominance (F:M ratio ∼9:1), our PTILN cohort demonstrated a nearly equal sex distribution (F:M = 9:10; Table 1). Furthermore, a substantial proportion (36.8%, 7/19) presented at ≥60 years of age. This demographic inversion—characterized by increased male representation and older age at presentation—poses significant diagnostic challenges, particularly when systemic lupus features are subtle or atypical. These distinct demographic features strongly imply that PTILN may arise from divergent immunopathogenic mechanisms or involve unique genetic susceptibility factors compared with glomerulocentric LN forms.

Consistent with prior observations [22], the renal presentation of PTILN is distinct from that of typical lupus nephritis. Subnephrotic-range proteinuria (median 0.76 g/24 h) or its absence predominated in our cohort (18/19 patients), with nephrotic syndrome documented in only a single historical case [12]. In stark contrast, AKI is the most common presenting feature, affecting 57.9% (11/19) of patients [7,8,10,11,14,15,19,20,23]. This pattern of significant functional impairment (median admission SCr 2.7 mg/dL, eGFR 20.27 mL/min/1.73 m^2^) with minimal proteinuria—exemplified by our patient’s presentation (SCr 916 μmol/L, UPCR 680 mg/g Cr)—strongly correlates with the primary histopathological lesion of severe tubulointerstitial inflammation and damage.

Despite its atypical renal presentation, our cohort universally demonstrated immunological evidence of SLE: 100% ANA positivity (18/18 evaluated), 94.1% (16/17) anti-dsDNA antibody positivity, and 93.3% (14/15) hypocomplementemia (Table 1 and Supplementary Table 4). These findings, coupled with the near-universal presence of hematologic abnormalities (100% of 17 evaluated patients exhibiting anemia, leukopenia, and/or thrombocytopenia), provide critical diagnostic clues. Consequently, PTILN must be actively considered in older male patients (≥60 years) presenting with AKI and subnephrotic-range proteinuria, even in the absence of classic glomerulonephritis features or overt extrarenal manifestations.

The defining histopathological triad of PTILN—minimal glomerular involvement, dominant tubulointerstitial damage, and TBM-predominant immune deposition—was consistently observed across all 19 patients (Table 2 and Supplementary Table 3): (1) Minimal glomerular involvement: All patients (100%) presented an absence of crescents, endocapillary proliferation, or significant sclerosis. (2) Dominant tubulointerstitial injury: 89.5% mononuclear infiltration, 68.4% fibrosis, and 63.2% tubular atrophy. Severe involvement (>50% parenchyma) was observed in 33.3% of patients with atrophy and 36.4% with fibrosis. (3) TBM-predominant immune deposits: Granular TBM deposits of IgG (93.3%), C3 (86.7%), and C1q (73.3%) were observed in 94.4% of the patients. Mesangial immune complexes were fainter (50%) and quantitatively subordinate to TBM deposits. Electron microscopy confirmed electron-dense deposits within the TBM (61.5%) or mesangium (46.2%). This TBM-centric immune complex deposition—contrasting sharply with glomerulocentric patterns in classic LN—supports an ‘*in situ*’ immune complex-driven pathogenesis.

We addressed clinical issues by integrating key insights from our cohort of 19 patients, contrasting PTILN with classic glomerular LN, and providing actionable clinical guidance. Our study reveals ‘four fundamental contrasts’ between PTILN and classic glomerular LN (see Table 4). These differences underscore PTILN as a demographically, clinically, and pathologically distinct variant of LN.

**Table 4.** The ‘four fundamental contrasts’ between PTILN and classic LN.

Feature	Classic glomerular LN	PTILN (our cohort)	Clinical implication
Demographics	9:1 Female predominance; younger onset (16–55 yrs)	Near-equal sex ratio (F:M = 9:10); 36.8% ≥60 yrs	Older males with AKI warrant PTILN suspicion despite atypical SLE presentation
Renal Presentation	Heavy proteinuria (nephrotic range) is common; gradual SCr rise	AKI-dominant (57.9%); subnephrotic proteinuria (median 0.76 g/24 h)	AKI + minimal proteinuria may mask SLE; avoid misdiagnosis as ATN/interstitial nephritis
Histopathology	Glomerulocentric (crescents, endocapillary proliferation)	Tubulointerstitial-dominant: TBM immune deposits (94.4%), mononuclear infiltration (89.5%), fibrosis (68.4%)	Biopsy essential: Glomerular-sparing pattern requires IF/EM for TBM deposits
Serological Clues	Anti-dsDNA+(80–90%), low C3/C4	Universal ANA+ (100%); anti-dsDNA+ (94.1%); low C3/C4 (93.3%)	Serology remains pivotal despite atypical renal presentation

SCr: serum creatinine; AKI: acute kidney injury; ATN: acute tubular necrosis; IF: immunofluorescence; EM: electron microscopy; TBM: tubular basement membrane.

PTILN requires rigorous exclusion of IgG4-RD, primary Sjögren’s syndrome (pSS), drug-induced renal damage, among others. Patients with overlapping features (pSS) were excluded per reference [25].

Given the rarity of PTILN, standardized therapeutic protocols remain undefined, with no clinical trials specifically evaluating treatment approaches for this variant. Current management is primarily extrapolated from general lupus nephritis principles. Our analysis revealed that glucocorticoids formed the therapeutic backbone in 88.9% (16/18) of patients, with monotherapy employed in 56.3% (9/16) of treated patients (Table 3 and Supplementary Table 3). Notably, combination immunosuppression has gained traction in recent years: mycophenolate mofetil has emerged as the most frequent adjunct (22.2%, 4/18), yielding favorable renal outcomes in all reported cases—including our patients—with sustained stability over 10–32 months of follow-up [7,21,23].

A notable finding was the limited documentation of HCQ use, reported in only 2 historical cases [7,23]. Our patient received HCQ alongside GC and MMF, highlighting a potential therapeutic evolution. This approach aligns with emerging evidence that HCQ may mitigate tubulointerstitial inflammation in LN by inhibiting Toll-like receptor signaling and autophagy-mediated damage [26]. Future studies should systematically evaluate whether the immunomodulatory properties of HCQ confer adjunctive benefits in PTILN pathogenesis.

Treatment outcomes were generally favorable but underscored significant prognostic implications. A robust 83.3% (15/18) of patients achieved complete or partial renal response. Only one case of mortality (5.6%) was documented—a 72-year-old male who presented with nephrotic-range proteinuria (7.9 g/day) and who succumbed to sepsis following delayed treatment initiation [12]. This patient highlights key predictors of poor outcomes in PTILN patients: advanced age (>70 years), heavy proteinuria (nephrotic range), infectious complications, and therapeutic delays—factors warranting aggressive intervention when present.

The patient predominantly manifested tubulointerstitial LN. The pathogenesis of PTILN remains poorly understood but appears distinct from that of classic LN. The hallmark of PTILN is the dominant deposition of immune complexes along the TBM, which contrasts with the glomerular-centric lesions observed in most LN subtypes. The presence of immune complex deposition on the TBM suggests a potential *in situ* formation process, possibly initiated by the binding of circulating autoantibodies to exogenous or endogenous antigens, which is particularly intriguing in cases where no obvious glomerular lesions are observed [7]. Circulating autoantibodies may bind to cryptic antigens exposed in the TBM, particularly in the setting of tubular injury or oxidative stress [27]. This hypothesis is supported by the presence of granular IgG and complement deposits along the TBM, as well as the detection of electron-dense deposits using electron microscopy.

Recent research has revealed the possibility of germinal centre-like structures forming within the renal interstitium of patients with LN, which might secrete autoantibodies, leading to the formation of immune complexes along the TBM, complement system activation, and subsequent development of focal inflammatory lesions and fibrosis [28]. However, neither our patient nor the patients described by Tan et al. [7] presented such structures in renal biopsies, highlighting the need for further investigation into the pathogenesis of this condition.

Autoantibody-producing plasma cells (PCs) play a central role in the pathogenesis of antibody-mediated autoimmune diseases, including RA and SLE [29,30]. With respect to SLE, PCs located in the nephritic kidneys are responsible for producing autoantibodies [31]. CD38 is a type II glycoprotein that is highly and uniformly expressed on antibody-producing plasmablasts and PCs. In recent years, increasing evidence has shown that the CD38 protein may play an indispensable role in autoimmune diseases [32,33].

Patients with SLE often exhibit altered CD38 expression, specifically at the level of distinct cell types [34]. In an *ex vivo* study of CD38 expression on various immune cells in peripheral blood mononuclear cells from patients with SLE, the highest CD38 expression was observed on PCs and plasmablasts [34]. In the renal tissues of this patient, immunohistochemical studies revealed multifocal infiltration of CD38-positive cells in the renal interstitium, suggesting a potential role in the pathogenesis of predominant tubulointerstitial LN. PCs infiltrating the interstitium may directly damage tubular epithelial cells through cytokine release or cytotoxic effects. The prominence of CD38-positive PCs in some cases suggests a role for dysregulated plasma cell activity in perpetuating inflammation. Further research with an expanded set of clinical cases and additional renal tissue samples is warranted to investigate this aspect more comprehensively.

In our patient, the patient received a combination of methylprednisolone and MMF for induction therapy, which yielded positive outcomes. The serological indicators of SLE activity remained stable for more than 2 years with a reduced glucocorticoid dosage. Currently, patients are being treated with a regimen of low-dose glucocorticoids and MMF without any relapse. As seen in the other three patients, the addition of MMF to glucocorticoid therapy demonstrated promising results [7,21,23], and early initiation of steroid and MMF therapy appears to be crucial in preventing disease progression and preserving renal function.

This study has several important limitations inherent to investigating rare diseases: 1. Limited cohort size: Despite a systematic global literature search, the rarity of PTILN restricted our cohort to 19 patients. This small sample size precludes robust statistical analyses (e.g., multivariate regression) and limits subgroup comparisons (e.g., treatment efficacy across demographic strata). 2. Retrospective data constraints: Our dependence on published case reports introduced heterogeneity in (a) data completeness (e.g., missing electron microscopy in 6/19 patients); (b) diagnostic standards (older cases lacked the 2018 ISN/RPS classification); and (c) outcome reporting (inconsistent follow-up duration/metrics). 3. Therapeutic heterogeneity: Treatment strategies evolved substantially over the 44-year reporting period (1976–2022), with variable immunosuppressive regimens and dosing. This precludes meaningful efficacy comparisons between therapies. 4. Pathology reclassification challenges: Attempts to uniformly apply the 2018 ISN/RPS criteria to historical biopsies were limited by incomplete staining protocols in older reports and evolving standards for tubulointerstitial lesion quantification. 5. Biomarker Gap: No studies have reported urinary biomarkers of tubular injury (e.g., N-acetyl-β-D-glucosaminidase, β2-microglobulin, and kidney injury molecule 1). This obscures potential correlations between biomarker dynamics and histopathological progression. These constraints collectively limit mechanistic insights and treatment recommendations and underscore an urgent need for prospective multicenter registries featuring protocolized biopsy processing (including IgG4/CD38 staining), standardized outcome assessments (e.g., serial eGFR, proteinuria, biomarkers), and centralized pathology review using current ISN/RPS criteria.

## 5. Conclusion

This study establishes PTILN as a distinct clinicopathological entity characterized by dominant tubulointerstitial damage with TBM immune deposits (IgG/C3/C1q), minimal glomerular involvement, demographic inversion (male predominance, elderly onset), and AKI-predominant presentation with subnephrotic proteinuria. Outcomes are generally favorable with respect to immunosuppression. PTILN warrants suspicion in elderly males with AKI and SLE serology. Future priorities include validated diagnostic criteria integrating TBM immune complexes, tubular biomarkers (e.g., NAG/β2MG), and targeted therapeutic trials. Multicenter registries are essential to optimize management.

## Supplementary Material

Supplemental Material

## Data Availability

The data of the clinical case are available from the corresponding author upon reasonable request. They will be provided anonymously.
