# Supplementary material for: Literature review and case study of predominant tubulointerstitial lupus nephritis
Source: Ren Fail. 2025 Sep 18;47(1):2558089. doi: 10.1080/0886022X.2025.2558089 (PMC12447453; doi:10.1080/0886022X.2025.2558089)
Supplement: Supplemental Material [file IRNF_A_2558089_SM6776.docx]

**Supplementary information**

**Supplementary Table 1.** The patient's laboratory data at admission.

| Laboratory data | Values | Reference range |
| --- | --- | --- |
| White blood cell (×10^9^/L) | 3.22 | 3.5-9.5 |
| Neutrophil ratio (%) | 68.8 | 40-75 |
| Hemoglobin (g/L) | 64 | 130-175 |
| Hematocrit (%) | 19.5 | 40-50 |
| Mean corpuscular volume (fL) | 89.9 | 82-100 |
| Platelet (×10^9^/L) | 56 | 125-350 |
| Absolute Reticulocyte Count((×10^12^/L) | 0.028 | 0.024-0.084 |
| Reticulocyte percentage（%） | 1.07 | 0.5-1.5 |
| Schistocytes (%) | Not identified | <0.5% in peripheral blood |
| Direct Coombs test | Negative | Negative |
| Total protein (g/L) | 64.7 | 65-85 |
| Albumin (g/L) | 29.5 | 40-55 |
| Total bilirubin（umol/l） | 3.5 | 0-26 |
| Indirect bilirubin（umol/l） | 2.5 | 0-18 |
| Lactate Dehydrogenase(U/L) | 213 | 120-250 |
| Haptoglobin(mg/dL) | 103.6 | 30-200 |
| Urea (mmol/L) | 39.6 | 3.6-9.5 |
| Creatinine (μmol/L) | 916 | 57-111 |
| Calcium (mmol/L) | 1.96 | 2.11-2.52 |
| Phosphorus (mmol/L) | 2.35 | 0.85-1.51 |
| Potassium (mmol/L) | 5.46 | 3.5-5.3 |
| Creatine kinase (U/L) | 534 | 50-310 |
| Alkaline phosphatase (U/L) | 50 | 45-125 |
| C-reactive protein (mg/L) | 6.7 | 0-5 |
| Urine pH | 6.0 | 4.6-8.0 |
| Urine protein | + | Negative |
| Urine latent blood | 2+ | Negative |
| Urine glucose | + | Negative |
| Urine RBC(/μL) | 6.4 | 0-14.4 |
| Urine RBC(/HPF) | 0-1 | 0-3 |
| Urine WBC(/HPF) | Negative | 0-5 |
| Urine WBC(/μL) | 1.3 | 0-10.1 |
| Hyaline casts(/LPF) | None seen | 0-1 |
| White blood cell casts(/LPF) | None seen | Negative |
| Red blood cell casts(/LPF) | None seen | Negative |
| Tubular epithelial cell casts(/LPF) | None seen | Negative |
| Granular casts(/LPF) | None seen | Negative |
| Urinary protein-to-creatinine ratio (mg/g Cr) | 680.13 | 0-164 |
| Complement C3(g/L) | 0.4 | 0.7-1.4 |
| Complement C4(g/L) | 0.09 | 0.1-0.4 |
| Complement C1q(mg/L) | 196 | 159-233 |
| Anti-double-stranded DNA antibody | + | Negative |
| Anti-Smith antibody | Negative | Negative |
| Anti-SS-A antibody | Negative | Negative |
| Anti-SS-A antibody | Negative | Negative |
| Anti-Jo-1 antibody | Negative | Negative |
| Anti-Ro-52 antibody | Negative | Negative |
| Anti-ribosomal P protein antibodies | Negative | Negative |
| Antinuclear antibody titers | 1:1000 | Negative |
| Anti-cardiolipin Ig (GAM) (AU/mL) | <5 | 0-20 |
| Anti-cardiolipin IgG (GPL-U/mL) | 3.97 | 0-10 |
| Anti-cardiolipin IgM (MPL-U/mL) | <2 | 0-10 |
| Anti-β₂-glycoprotein I Ig (GAM) (AU/mL) | 4.2 | 0-5 |
| Lupus anticoagulant Screening Test(s) | 41.2 | 31-44 |
| Lupus anticoagulant Confirmatory Test(s) | 37.9 | 30-38 |
| Lupus anticoagulant Ratio | 1.09 | 0.8-1.2 |
| MPO-ANCA | Negative | Negative |
| PR3-ANCA | Negative | Negative |
| Anti-glomerular basement membrane antibodies | Negative | Negative |
| Rheumatoid factor (IU/mL) | 123 | 0-16 |
| Anti-cyclic citrullinated peptide antibody (U/mL) | Negative | Negative |
| Light chain κ/λ | 1.95 | 1.17-2.93 |
| Hepatitis B surface antigen (IU/mL) | 0 | <0.05 |
| Hepatitis C antibody(S/CO) | 0.12 | <1 |
| Serum IgG levels(g/L) | 23.32 | 8.6-17.4 |
| Serum IgG4 levels(g/L) | 1.2 | 0.08-1.51 |
| Serum IgA levels(g/L) | 1.49 | 1.0-4.2 |
| Serum IgM levels(g/L) | 1.26 | 0.5-2.8 |
| Serum IgE levels (IU/mL) | 205 | 0-358 |

**Supplementary Table 2.** Integrated treatment timeline and renal function evolution.

| **Timeline** | SCr **(μmol/L)** | **UPCR (mg/g Cr)** | **Medication Regimen (Dosage)** |
| --- | --- | --- | --- |
| **Baseline** | 916 | 680 | **Methylprednisolone**:250 mg IV ×3 days → 40 mg IV daily  **MMF**: Initiated at 2 g/day  **HCQ**: 400 mg/day |
| **Month 1** | 428 | 502 | **Methylprednisolone**: 40 mg IV daily → transition to oral  **MMF**: 2 g/day maintained  **HCQ**: 400 mg/day |
| **Month 2** | 311 | 371 | **Dialysis discontinued**  **Methylprednisolone:** Reduced to 20mg/day, then tapered by 4mg every 2 weeks  **MMF:** 2 g/day  **HCQ**: 400 mg/day |
| **Month 3** | 332 | 262 | **Methylprednisolone**: Reduced to 12mg/day, then tapered by 2mg every 2 weeks  **MMF**: 2 g/day  **HCQ**: Reduced to 300 mg/day |
| **Month 4** | 307 | 231 | **Methylprednisolone:** Tapered by 2mg every 2 weeks, reduced to 4 mg/day oral  **MMF:** 2 g/day  **HCQ:** 300 mg/day |
| **Month 5** | 286 | 357 | **Methylprednisolone:** 4 mg/day maintained  **MMF:** 2 g/day  **HCQ:** 300 mg/day |
| **Month 6** | 292 | 220 | **Methylprednisolone**: 4 mg/day  **MMF**: Reduced to 1.5 g/day  **HCQ**: Reduced to 200 mg/day |
| **Month 9** | 277 | 285 | **Methylprednisolone:** 4 mg/day  **MMF:** Reduced to 1.25 g/day  **HCQ:** 200 mg/day maintained |
| **Month 12** | 233 | 221 | **Methylprednisolone:** 4 mg/day  **MMF:** Reduced to 1.0 g/day  **HCQ:** 200 mg/day |
| **Month 18** | 226 | 203 | **Methylprednisolone:** 4 mg/day  **MMF:** 1.0 g/day maintained  **HCQ:** 200 mg/day |
| **Month 24+** | 200-300 | 179-307 | **Maintenance**:  **Methylprednisolone:** 4 mg/day  **MMF:** 1.0 g/day  **HCQ:** 200 mg/day |

SCr: Serum creatinine, UPCR: Urinary protein-to-creatinine ratio, MMF: Mycophenolate mofetil, HCQ: Hydroxychloroquine

**Supplementary Table** **3.** Renal manifestations of literature-reported cases of PTILN.

| No. of  case | Age  (yrs) | Sex | Presentation | Urine analysis | LM | IF | EM | Therapy and prognosis |
| --- | --- | --- | --- | --- | --- | --- | --- | --- |
| 1 [8] | 52 | F | AKI | 3+ protein | Interstitial fibrosis and mononuclear cell infiltration; tubular atrophy; glomeruli appeared almost normal | Granular IgG, IgM, C3 in TBM; faint C3 in mesangium | EDD in TBM | Corticosteroid+ AZA (PR) |
| 2 [9] | 23 | F | Tubular acidosis | No proteinuria | Focal interstitial fibrosis and tubular atrophy; normal glomeruli | C3 in TBM; negative in glomeruli | (-) | Corticosteroid (To be unaffected by corticosteroid therapy, CR) |
| 3 [10] | 30 | F | AKI | No proteinuria | Mononuclear cell infiltration; tubular atrophy; mild mesangial cell proliferation | Granular IgG, C3 in TBM; interstitium, mesangium | EDD in mesangium | Corticosteroid (CR) |
| 4 [11] | 42 | F | AKI | Mild proteinuria | Interstitial fibrosis; lymphocytes and plasma cells infiltration; mild and focal mesangial cell proliferation | Granular IgG, Clq in TBM; small IgG, Clq, C3 in mesangium | (-) | Corticosteroid (PR) |
| 5 [11] | 24 | F | AKI | Proteinuria | Diffuse interstitial fibrosis and moderate mononuclear cell infiltration; ischemic glomeruli; mild mesangial cell proliferation | Granular IgG, C1q, C3, IgM in TBM; negative in glomeruli | (-) | Peritoneal dialysis, neither steroid nor immunosuppressive drug was given (ESRD) |
| 6 [12] | 72 | M | RI + NS | 4+ protein | Focal, chronic interstitial inflammation and fibrosis; minimal change in glomeruli | IgG, IgM, C3 in interstitium | Fusion of foot process | Corticosteroid and cyclophosphamide (death from sepsis) |
| 7 [13] | 3 | M | Without RI | 1+ protein | Interstitial fibrosis and mononuclear cell infiltration; tubular atrophy; mild increase in mesangial cell and matrix | Linear IgG in TBM and Bowman's capsule; granular IgG, IgA, C3，C4, C1q in mesangium | EDD in mesangium, not in TBM | Corticosteroid (CR) |
| 8 [14] | 25 | F | AKI | 1+ protein | Mononuclear cell infiltration; tubular atrophy; minimal change in glomeruli | IgG in Bowman's capsule; C3 in TBM; mild C3 in mesangium | No EDD | Supportive therapy  (NR) |
| 9 [15] | 59 | M | AKI | Mild  proteinuria | Interstitial fibrosis and mixed cellular infiltration; tubular atrophy; normal glomeruli | Granular IgG, IgA, IgM, C3, C1q in TBM, interstitium, and Bowman's capsule; negative in glomeruli | EDD in TBM and Bowman's capsule | Corticosteroid  (PR) |
| 10 [16] | 30 | F | RI | Mild  proteinuria | Interstitial edematous and mononuclear cell infiltration; tubular atrophy; minimal change in most glomeruli | Granular IgG, C3, C1q in TBM | (-) | Corticosteroid  (PR) |
| 11 [17] | 64 | M | RI | Mild  proteinuria | Interstitial fibrosis and mononuclear cell infiltration; tubular atrophy; minimal change in glomeruli | Granular IgG, C3, C1q in TBM | EDD in TBM | Corticosteroid  (CR) |
| 12 [18] | 63 | M | Without RI | No proteinuria | Interstitial fibrosis and mononuclear cell infiltration; tubular atrophy; mild increase in mesangial cell | Granular IgG, IgA, IgM, κ, λ, C3, C1q in TBM, mesangium and peritubular interstitium | EDD in the mesangium and in TBM | Corticosteroid (CR) |
| 13 [19] | 67 | F | AKI | Mild  proteinuria | Focal tubular atrophy, abundant lymphocyte infiltrates, mostly from plasma cells. No significant glomerular alterations | Granular deposits in the arterial walls of C3 and tubular cylinders of IgA | (-) | Corticosteroid  (Not mentioned) |
| 14 [24] | 38 | M | AKI | Mild  proteinuria | Interstitial mononuclear cell infiltration; normal glomeruli | IgG, IgA, Clq, C3in TBM and peritubular capillary basement membranes; negative in glomeruli | (-) | Corticosteroid + AZA  (CR) |
| 15 [20] | 18 | F | RI | Mild  proteinuria | Diffuse and focally destructive, focally enhanced tubulointerstitial inflammatory infiltrate, Minimal glomerular changes with slight mesangial hypercellularity | - | Mesangial deposits | Corticosteroid and  Cyclophosphamide/MMF (PR) |
| 16 [21] | 72 | M | RI | Mild  proteinuria | Focal tubular atrophy and interstitial fibrosis; Interstitial mono-nuclear infiltration; lymphoid follicle in the interstitium; normal glomeruli | IgG and C1q in TBM;  negative in glomeruli | EDD in TBM | Corticosteroid  (PR) |
| 17 [7] | 67 | M | AKI | 1+ protein | Tubular epithelial cells exhibited focal vacuolization and eosinophilic granules in the cytoplasm and focal loss of brush border. Interstitial lymphocytes, plasma cells, and a few eosinophils infiltration. Interstitial "Storiform" fibrosis with tubular atrophy. Partial glomeruli were globally sclerosed and ischemic sclerosis. Other glomeruli showed no significant glomerular change. | IgG, C3, Clq in TBM, interstitium, mesangium | EDD in TBM and mesangium | Corticosteroid and MMF+HCQ  (PR) |
| 18 [22] | 39 | F | AKI | 1+ protein | Mild foci of interstitial fibrosis and tubular atrophy; granular fuchsinophilic hyaline deposits in TBM. Minority glomeruli were globally sclerotic, and the remaining were normal; no signs of interstitial inflammation | IgG, C3, C1q, κ, λ in TBM; IgG with mild positivity in mesangium | EDD in TBM | Corticosteroid and MMF+HCQ  (PR) |
| 19(OUR CASE) | 64 | M | AKI | 1+ protein | Interstitial fibrosis and mononuclear cell infiltration; tubular atrophy; minimal change in glomeruli | IgG, C3, C1q, κ, λ in TBM, mesangium | EDD in TBM and mesangium | Corticosteroid and MMF+HCQ (PR) |

AKI: Acute Kidney Injury, RI: Renal Impairment, NS: Nephrotic Syndrome, LM: Light Microscopy, IF: Immunofluorescence, EM: Electron Microscopy, TBM: Tubular Basement Membrane, EDD: Electron Dense Deposits, κ: Kappa, λ: Lambda. CR: Complete Response, PR: Partial Response, NR: No response. Numbers in brackets correspond to reference numbers, (-) represents no electron microscope results.

**Supplementary Table 4.** Extrarenal manifestations and biological features of literature-reported cases of PTILN.

| No. of  case | Age  (yrs) | Sex | Constitutional (Fever) | Hematologic | Neuropsychiatric | Mucocutaneous | Serosal | Musculoskeletal | Complement | Specific antibodies | EULAR/ACR 2019 SLE Criteria |
| --- | --- | --- | --- | --- | --- | --- | --- | --- | --- | --- | --- |
| 1 [8] | 52 | F | (+) | Anemia | Epilepsy and somnolence | Not mentioned | (+) | Polyarthritis | C3 53 mg/dL ↓ | ANA (+)  L.E.-cell test(+) | 31 |
| 2 [9] | 23 | F | Not mentioned | Not mentioned | Encephalitis | Malar rash | Not mentioned | Polyarthritis | Not mentioned | Serologically confirmed (Not mentioned) | 19 |
| 3 [10] | 30 | F | (+) | Anemia | Not mentioned | Not mentioned | Not mentioned | Joint tenderness | CH50 34 u/L Not mentioned C3 or C4 | ANA 1:640 (+)  Anti-nRNP (+)  anti-Sm (+) anti-dsDNA (+)  anti-dsDNA (+) | 16 |
| 4 [11] | 42 | F | (+) | Anemia | Not mentioned | Malar rash | (+) | Arthritis | C3 24 mg/dL↓  C4 18 mg/dL | ANA (+)  L.E.-cell test (+)  Serum DNA binding 90% | 25 |
| 5 [11] | 24 | F | (+) | Anemia | Not mentioned | Not mentioned | (+) | Joint tenderness | C3 104 mg/dL  C4 21 mg/dL | ANA (+)  L.E.-cell test (+)  Serum DNA binding 95% | 22 |
| 6 [12] | 72 | M | Not mentioned | Anemia | (-) | Not mentioned | (+) | Not mentioned | C3 53 mg/dL↓  C4 9 mg/dL↓ | ANA 1:2560 (+)  anti-DNA (+) | 24 |
| 7 [13] | 3 | M | (+) | Anemia  Thrombocytopenia  Leukopenia | Not mentioned | papular and purpuric skin lesions on the limbs, trunk, and face. | Not mentioned | Not mentioned | C3 46 mg/dL↓  C4 6.4 mg/dL↓ | ANA 1:160 (+)  L.E.-cell test (+)  anti-DNA (+++) | 26 |
| 8 [14] | 25 | F | (+) | Anemia | Not mentioned | Morbilliform rash (face, trunk); mucosal rash; Koplik’s spots | (+) | Polyarthritis | C3 33 mg/dL↓ | ANA 1:320 (+++)  anti-dsDNA：3.4µg DNA/ml↑  anti-SM （+） | 37 |
| 9 [15] | 59 | M | (+) | Anemia | Not mentioned | Photosensitive facial rash | Not mentioned | Knee pain | C3 0.54 mg/mL↓  C4 <0.008 mg/mL↓  CH50 < 10U/L↓ | ANA 1:640 (+)  anti-dsDNA:0.6 | 26 |
| 10 [16] | 30 | F | (+) | Anemia  Thrombocytopenia  leukopenia | Not mentioned | Malar rash | Not mentioned | Pain in small joints | C3 46 mg/mL↓  C4 13 mg/mL | ANA 1:640 (+)  anti-dsDNA:36U/dL↑  Anti-Ro (+) | 25 |
| 11 [17] | 64 | M | (-) | Anemia | Not mentioned | Photosensitive facial rash | Not mentioned | Not mentioned | C3 42 mg/dL↓  C4 4 mg/dL↓  CH50 < 10U/L↓  IC(Clq) 9.1μg/ml↑ | ANA 1:640 (+)  anti-dsDNA :525U/ml↑ | 24 |
| 12 [18] | 63 | M | Not mentioned | Thrombocytopenia | Not mentioned | Not mentioned | Not mentioned | Polyarthralgia | C3 24 mg/dL↓  C4 7 mg/dL↓  CH50 11U/mL↓  IC(Clq) 6.8μg/ml↑ | ANA 1:280 (+)  anti-dsDNA:31.4U/ml↑ | 20 |
| 13 [19] | 67 | F | Not mentioned | Anemia | Not mentioned | Not mentioned | Not mentioned | Not mentioned | Not mentioned | ANA (+)  anti-dsDNA (+) | 14 |
| 14 [24] | 38 | M | (-) | Anemia  leukopenia | Not mentioned | Malar rash | Not mentioned | Polyarthralgia | C3 31 mg/dL↓  C4 14 mg/dL↓ | ANA (+)  anti-dsDNA (+) | 30 |
| 15 [20] | 18 | F | Not mentioned | Not mentioned | Not mentioned | Not mentioned | Not mentioned | Polyarthralgia | C3 0.77 g/L↓  C4 0.121 g/L | ANA 1:5120(+)  anti-dsDNA 201 U/ml↑ | 19 |
| 16 [21] | 72 | M | (-) | Anemia  Thrombocytopenia  Leukopenia | (-) | (-) | (-) | Polyarthritis | C3 40 mg/dL↓  C4 2 mg/dL↓  CH50 < 12U/L↓ | ANA 1:320(+)  anti-dsDNA:298 IU/ml↑ | 24 |
| 17 [7] | 67 | M | (-) | Anemia | Not mentioned | Photosensitive facial rash | Not mentioned | Not mentioned | C3 0.24 g/L↓  C4 <0.017 g/L↓ | ANA 1:10000(+)  anti-dsDNA>800 IU/ml↑ | 24 |
| 18 [22] | 39 | F | (-) | leukopenia | Not mentioned | Malar rash | （-） | Polyarticular synovitis | Not mentioned | ANA (+)  anti-dsDNA (+) | 25 |
| 19  (OUR CASE) | 64 | M | (-) | Anemia  Thrombocytopenia  leukopenia | (-) | (-) | (-) | (-) | C3 0.4 g/L↓  C4 <0.09 g/L↓ | ANA 1:1000(+)  anti-dsDNA (+) | 18 |

(+) represents relevant clinical manifestations, (-) represents no relevant clinical manifestations, ↑ represents greater than the reference range, ↓ represents less than the reference range.
